# Comparison Between Disability, Physical Performance, and Other Biopsychosocial Factors in Full-Duty Career Firefighters Working With and Without Current Low Back Pain

**DOI:** 10.7759/cureus.62189

**Published:** 2024-06-11

**Authors:** John M Mayer, Joe L Verna, Michael Hubka, Brandon Phelps, Chris Wolfinger, Charity L Lane

**Affiliations:** 1 Research, The Vert Mooney Research Foundation, San Diego, USA; 2 Clinical, Spine & Sport Physical Therapy, San Diego, USA

**Keywords:** fitness, wellness, biopsychosocial model, work readiness, injuries, low back pain, firefighters

## Abstract

Introduction: Low back pain (LBP) is highly prevalent and a top cause of disability-related early retirement in firefighters. Those with a lifetime history of LBP have various deficiencies that are associated with increased injury risk and absenteeism. However, the influence of working with current LBP on disability, physical performance, and other biopsychosocial factors has not been fully characterized in this population. The purpose of this study was to compare anthropometric measures, exercise habits, physical fitness/performance, disability/work ability, and other biopsychosocial factors of firefighters working with and without current LBP.

Methods: A cross-sectional study was conducted using baseline assessments from 419 full-duty career firefighters without documented work restrictions (age: 37.6 ± 8.8 y; 5 F, 414 M) who were enrolled in a regional wellness initiative in Southern California, USA. Current LBP status was determined by a questionnaire and confirmed by an interview. Anthropometric measures, patient-reported outcomes, and physical fitness tests were used to assess body mass index; body fat %; waist circumference; strengthening, cardiovascular, and flexibility exercise frequency; back and core muscular endurance; functional movement quality, perceived back-related disability, lift and carry ability, and firefighter task ability; sleep quality; and perceptions of fear and fatigue and catastrophic injustice experience. Scores for participants with and without current LBP were compared using analysis of variance and chi-square analysis.

Results: The point prevalence of current LBP was 19.81% (83/419). For the entire cohort, those with current LBP had significantly worse scores than those without current LBP for all assessed variables, except core muscular endurance and functional movement quality. These trends held up when analyses were stratified by age and obesity categories, and approximately half of the comparisons retained statistical significance. A significantly greater percentage of participants with current LBP were working with some level of back-related disability and/or perceived physical demand characteristics of work level below the required very heavy job demands.

Conclusion: Nearly one-fifth of full-duty career firefighters without documented work restrictions reported having current LBP, and these individuals had deficits in several modifiable biopsychosocial factors across five health domains. These findings can help guide future research and implementation efforts in the fire service designed to improve performance, resiliency, work readiness, recovery, and quality of life, as well as to reduce impairment, disability, and absenteeism and increase presenteeism.

## Introduction

Musculoskeletal injuries and associated chronic pain syndromes are remarkably burdensome in firefighters, greatly impacting the quality of life, physical and behavioral health, resiliency, and preparedness [1]. Of the musculoskeletal disorders, low back pain (LBP) is particularly problematic and a top cause of disability-related early retirement [1,2]. The lifetime prevalence of LBP in career firefighters is high, with published studies reporting prevalence rates ranging from 45% [3], 66% [4], to 86% [1]. Among the various physical and psychological job stressors encountered by these first responders, their demanding work environment is linked to musculoskeletal injuries [1]. Several work-related tasks have been specifically associated with an increased risk of LBP, such as "cut walls/ceiling/floors" (odds ratio (OR): 6.47), "operate charged house inside a building" (OR: 3.26), "climb ground/aerial ladders" (OR: 3.18), and "lift/carry objects > 18 kg" (OR: 3.07) [5].

Firefighters with a lifetime history of LBP and related health disorders have deficiencies in numerous biopsychosocial factors that are associated with increased injury risk and absenteeism. For example, those with a lifetime history of LBP are less physically fit and have poorer % body fat, aerobic capacity, back extension muscular endurance, and extremity strength [3]. Moreover, substandard lifting strength and physical fitness are associated with an increased risk of back injuries in this occupation [6]. Furthermore, firefighters with obesity are more likely to experience a musculoskeletal injury [7], have poor levels of back and core muscular fitness placing them at a great risk for low back injury [8], miss more work due to injury and have higher costs related to absenteeism [9], have higher rates of work-related disability [10], and are more likely to file a workers' compensation claim due to injury [11]. Firefighters are also at high risk for poor sleep [12], as well as behavioral health conditions, such as depression, posttraumatic stress disorder (PTSD), and suicide ideation [13], both of which can contribute to LBP [14,15].

In addition to the high prevalence of lifetime history of LBP, many firefighters report having current LBP, with published point prevalence rates ranging from 30.2% [16], 43.9% [17], to 55% [1]. Previous work has identified that current LBP in this population is related to fear avoidance behaviors and disability [17], stress, age, and body mass index [16], but not general fitness [16]. These preliminary studies are useful to help guide future research, yet they have some limitations. For example, it is unknown if the firefighters in the aforementioned studies were working full duty without documented work restrictions at the time of assessments. Furthermore, a wide range of biopsychosocial factors that could be important to LBP were not concurrently assessed, such as back muscular endurance, perceived work readiness, lifting ability, and sleep quality.

While the history of LBP is a strong predictor of future LBP [18], it is possible that current LBP is more valuable for clinical decision-making and guiding interventions aimed at injury prevention and treatment. However, more research is needed to fully characterize how working with current LBP influences important modifiable biopsychosocial risk factors, particularly those related to disability, performance, injury, work readiness, resiliency, and recovery in full-duty career firefighters without documented work restrictions.

Firefighting is multifactorial, physically and psychologically demanding, and requires high levels of knowledge, skills, and experiences [1,19]. Thus, the biopsychosocial model of prevention and treatment could be particularly useful for firefighters. This model of care has been advocated for the management of musculoskeletal disorders, such as LBP [20], and "evaluates the integrated 'whole person', with both the mind and the body together as interconnected entities, recognizing biological, psychological, and social components of pain and illness" [21]. In the biopsychosocial model, musculoskeletal pain and physical deconditioning interact with each other, make the other worse, and lead to psychological deconditioning, which lowers self-confidence, creates fear avoidance behaviors, and exacerbates physical deconditioning [21,22]. To adequately address these factors and their interconnections, clinically meaningful assessments across important health domains are beneficial, which in turn are used to guide the delivery of evidence-based and patient-centered interventions.

Some attempts at implementing interventions to address modifiable biopsychosocial risk factors in firefighters have been successful over the past decade [23]. However, we believe that barriers, fragmented access, and lack of a comprehensive biopsychosocial approach across prevention, clinical care, and resilience development have limited widespread adoption and long-term effectiveness. The purpose of this study was to compare anthropometric measures, exercise habits, physical fitness/performance, disability/work ability, and other biopsychosocial factors of full-duty career firefighters working with and without current LBP.

## Materials and methods

Study design

A cross-sectional study was conducted using baseline assessments encompassing various health domains obtained from participants who were enrolled in a regional firefighter wellness initiative [24]. The initiative was a quality improvement project consisting of an 18-month worksite health program targeting three conditions (musculoskeletal injuries, obesity, and sleep disorders). Its three-pronged approach consisted of screening and assessing, stratifying and triaging according to risk, and delivering multi-faceted interventions that were customized for individual goals and risk levels [24,25]. Its framework was generally aligned with the occupational health, wellness, and fitness recommendations of the National Fire Protection Association and the Fire Service Joint Labor Management Wellness Fitness Initiative [1,26,27].

Setting

Participants were employees of 15 fire departments in San Diego, San Bernardino, and Imperial Counties, California, USA. Recruitment and enrollment took place at a site within their department. 58.4% (244/419) of participants completed assessments while on-duty at a fire service site, and the others completed assessments while off-duty at a fire service site or an external clinical site. The intent was for all participants to complete the same assessments and for each participant to complete all assessments in one day.

The fire departments were generally small to medium in size (cumulatively employing approximately 900-1000 career firefighters) and included various types and jurisdictions, such as municipalities, fire protection districts, and reservations. First responders in these settings provided emergency responses throughout a wide range of areas, such as urban, wildland, and wildland-urban interface settings. Shift schedules varied as well, including 48 hours on/96 hours off, 4s and 6s, and strike force duties. Most of the departments required a physical ability test before hire, such as the Candidate Physical Ability Test (CPAT) [28], and none required a follow-up physical ability test post hire.

Participants

A convenience sample of 419 career firefighters (age: 37.6 ± 8.8 y; 5 F, 414 M) participated in this study. The inclusion criteria were career firefighters from the departments described above and full-duty employment, without documented work restrictions. The exclusion criteria were current workers’ compensation or personal injury case that was filed in the past 30 days or has not reached maximum medical improvement and was relevant to the project’s assessments; pregnant; and any other reason that, in the opinion of the principal investigator, would put the candidate at increased safety risk or otherwise make the candidate unsuitable for this study. For the analyses of the baseline assessment data described in this manuscript, additional exclusion criteria were fire chief; and missing data for current LBP from questionnaires or interviews.

Candidates were recruited by word of mouth, posted flyers, and face-to-face presentations by study staff. All candidates provided written informed consent prior to participation in this study. This study was reviewed and approved by Aspire IRB of the WCG IRB (Puyallup, Washington, USA) (IRB protocol number: 520190022).

Sample Size

Enrollment consisted of a sample of convenience in which all eligible candidates were invited to participate in the study that included completion of the baseline assessments described herein. Moreover, the over-arching grant from which these data were obtained was a quality improvement project focusing on implementation. Hence, a formal sample size calculation and power analysis were not conducted.

Tests and measures

Enrolled participants completed the assessments described below, which were coordinated or administered by study staff and included self-report electronic questionnaires (or paper in cases of IT issues), clinical interviews, anthropometric measures, and physical fitness/performance tests.

Independent Variable

The independent variable was the point prevalence of current LBP [29], which was determined by an item within a self-reported multidimensional health history questionnaire that was electronically administered via RedCap [30]. Responses were confirmed via interview with a licensed healthcare provider (i.e., chiropractor or physical therapist). The specific question was "Do you currently have low back pain?" [4,31,32], for which the dichotomous response (no, yes) was used for analysis. To clearly define the low back region, this question was accompanied by a visual diagram of the body with the low back region shaded, as well as a written description, "Low back pain refers to pain or other discomfort in the body region below the rib cage and above the lower buttocks." This description is consistent with other definitions [29,33], and this strategy has been used in other research on back injuries in firefighters [4,31].

Dependent Variables

The dependent variables consisted of assessments across five health domains: anthropometric, exercise habits, physical fitness/performance, disability/work ability, and other biopsychosocial. These assessments were selected because of their relevance to firefighters, they are potential risk factors for LBP and musculoskeletal injuries, and they are valid and pragmatic tests that can be implemented in a variety of settings. Moreover, the comprehensive and multi-faceted nature of the assessments fits the need for various inputs to make decisions about triage, interventions, work ability, and return to work, which is particularly important within the biopsychosocial model for occupational injury management.

Anthropometric Measures

Body mass index (BMI): Body mass (kg) and height (cm) were assessed by a scale and stadiometer, respectively, and BMI was calculated from these values [34]. The raw value for BMI (m/kg^2^) and derived categories (not obese < 30 m/kg^2^, obese ≥ 30 m/kg^2^) [34] were used for analysis.

Body fat percentage: It was assessed by bioelectrical impedance analysis [34], using the hand-to-foot method with the Tanita Segmental Body Composition Monitor (Tanita, Arlington Heights, IL) and procedures outlined by the manufacturer. The raw value (% body fat) was used for analysis.

Waist circumference: It was assessed by a flexible tape measure, using procedures outlined for firefighters [1]. Three measurements were taken, and the average value of the three measurements (in cm) was used for analysis [34].

Exercise habits: Frequencies of strengthening, cardiovascular, and flexibility exercises were assessed by a self-report questionnaire. For each exercise type, the participant is asked to indicate how many days per week - on average - they performed the specific exercise over the past 90 days. The raw value (d/wk) was used for analysis. Exercise frequency information is used in accordance with the recommendations of the American College of Sports Medicine [34].

Physical Fitness/Performance Measures

Back muscular endurance: It was assessed by the Ito test [35], which is a physical fitness test where the participant is in a prone position on a mat, lifts the unsupported upper body parallel to the ground, and holds this position for as long as possible. The raw value for isometric hold time (sec) was used for analysis. The Ito test was selected because it requires no equipment, it can distinguish between those with and without LBP [35], and isometric back muscular endurance is related to the future incidence of LBP [36].

Core muscular endurance: It was assessed by the prone plank test [37], which is a physical fitness test where the participant is in a prone position on a mat, lifts the unsupported torso of the ground with the upper and lower body in straight alignment, and holds this position for as long as possible. The raw value for isometric hold time (sec) was used for analysis. The prone plank test was selected because it requires no equipment, is recommended for firefighters [1], and has been used with the target population [31].

Functional movement quality: It was assessed by four tests of the functional movement screen (FMS) [38]: deep squat, hurdle step, in-line lunge, and active straight leg raise. For each test, an evaluator rates the quality of performance on a scale from 0 to 3. The sum of four tests results in a total possible score of 0-12 (higher score indicates better movement quality), which was used for analysis. The FMS was selected because it is linked to musculoskeletal injury risk in firefighters [39] and can be used to guide exercise interventions [38].

Disability/Work Ability Measures

Back-related disability: It was assessed by the original Oswestry Disability Index (ODI) [40], which is a self-report questionnaire consisting of 10 items examining the impact of back disability on pain intensity, personal care, lifting, walking, sitting, standing, sleeping, sex, social, and travel. For each item, the participant selects a statement (from six total) that reflects their situation. An overall score is calculated ranging from 0% to 100% disability, which is categorized as follows: 0% No disability, 1-20% Minimal disability, 21-40% Moderate disability, 41-60% Severe disability, 61-80% Very severe disability, and 81-100% Complete disability. The percent and category scores were used for analysis. The ODI was selected because it reflects components of disability, physical function, and pain and is widely used and validated for LBP [40].

Lift and carry ability, work ability - perceived physical demand characteristics of work (PDC) level: They were assessed based on the Multidimensional Task Ability Profile (MTAP) [41,42], which is a 50-item self-report pictorial activity and task sort that uses pictures and captions to depict progressively demanding functional tasks of daily living and work. For each item, the participant rates their ability to complete a task on a 5-point scale: able, slightly restricted, restricted, very restricted, and unable, along with the "Don't Know" option. Lift and carry ability was assessed by a sub-set of 14 MTAP items focusing on lifting and carrying, for which the sum results in a total possible score of 0-56 (a higher score indicates higher functional ability) that was used for analysis. Work ability - perceived PDC level was assessed by the full 50-item version of the instrument and the PDC categories derived from its 0-200 total score: ≤ 37 Below sedentary, 38-99 Sedentary, 100-163 Light, 164-191 Medium, 192-199 Heavy, and 200 Very Heavy. The MTAP was selected because it is strongly correlated to functional capacity evaluation [41], linked to the U.S. Department of Labor's PDC of work [19], and a validated measure of perceived functional ability [41,42].

Firefighter functional task ability: It was assessed by the Firefighter Functional Task Questionnaire (FFTQ) [43], which is a 12-item developmental self-report questionnaire that evaluates the ability to perform common firefighter tasks. Ten items are modeled after CPAT events [28], including stair climb, hose drag, equipment carry, ladder raise, ladder extension, forcible entry, search, rescue, ceiling breach, and ceiling pull. Two items are more physically demanding tasks involving moving heavy victims - confined rescue and load stretcher onto the ambulance. For each item, the participant is asked to describe their current ability to perform the task on the 5-point rating scale: 0 Unable, 1 Severe difficulty, 2 Moderate difficulty, 3 Mild difficulty, and 4 No difficulty. The sum of 12 items results in a total possible score of 0-48 (a higher score indicates higher task ability), which was used for analysis. Information gleaned from the FFTQ is used to help customize exercise programs to match job demands, which is aligned with previous work [44].

Other Biopsychosocial Measures

Sleep quality: It was assessed by the Pittsburgh Sleep Quality Index (PSQI) self-report questionnaire [45]. The PSQI consists of nine items, some of which have multiple sub-components. Some items are verbal rating scales about sleep quality and patterns, and others ask for the time of day or quantity of sleep. From these items, a standardized scoring strategy is applied that results in a total possible score of 0-21 (a higher score indicates worse sleep quality), which was used for analysis. The PSQI was selected because it is a validated measure of sleep quality [45].

Fear and fatigue perception: It was assessed by the Fear and Fatigue Questionnaire (FAFQ) [46], which is a 10-item self-report questionnaire that evaluates fear avoidance and fear of symptom exacerbation that was adapted from previous work on fear of movement and fear of re-injury. For each item, the participant rates how a particular scenario affects their health or mental health condition on a three-point scale: 0 Do not agree, 1 Somewhat agree, and 2 Completely agree. For this study, the sum of 10 items was calculated resulting in a total possible score of 0-20 (a higher score indicates worse fear and fatigue), which was used for analysis. The FAFQ was selected because its information is useful for work disability management [46].

Catastrophic thinking and perceived injustice: It was assessed by the Catastrophic Injustice Experience Questionnaire (CIEQ) [47,48], which is a 12-item self-report questionnaire that evaluates catastrophizing and perceived injustice that was adapted from the Pain Catastrophizing Scale [47], and Injustice Experiences Questionnaire [48]. For each item, the participant indicates how frequently specific thoughts and feelings occur when they think about their health or mental health condition on a three-point scale: 0 Never, 1 Sometimes, and 2 Often. For this study, the sum of 12 items was calculated resulting in a total possible score of 0-24 (a higher score indicates worse catastrophic thinking and perceived injustice). The CIEQ was selected because its information is valuable for work disability management [49].

Analysis

Descriptive statistics were calculated, including means and standard deviations for continuous variables and frequencies (percent) for categorical variables. For the continuous dependent variables, comparisons between the two groups (i.e., those with and without current LBP) were made using one-way analysis of variance (ANOVA), which was the study's primary aim. Since this study was exploratory in nature and predicting current LBP from a set of other variables was not its aim, multiple regression or similar analysis was not conducted. Moreover, we hypothesized that numerous interrelationships would be present among the dependent variables, which precluded the use of analysis of covariance. In addition to comparing those with and without current LBP for the entire sample, the same analyses were conducted for categories of age (< 45 yr, ≥ 45 yr) and obesity (BMI < 30 kg/m^2^ - not obese, BMI ≥ 30 kg/m^2^ - obese), for which relationships with current LBP were anticipated. This approach was adapted from a recent cross-sectional study with firefighters examining a different independent variable [50].

For the categorical dependent variables, comparisons between the two groups (i.e., those with and without current LBP) were made using chi-square analysis. On an exploratory basis, the continuous dependent variables were compared by calculating the Pearson product-moment correlation coefficient (R) for each pairwise comparison, with the strength of correlation for absolute values of R as follows [51]: 0-0.19 Very weak, 0.20-0.39 Weak, 0.40-0.59 Moderate, 0.60-0.79 Strong, and 0.80-1.00 Very strong. Statistical significance was set at alpha = 0.05 for all analyses.

## Results

A total of 580 candidates were recruited and preliminarily screened for participation in this study, of which 151 were excluded prior to baseline assessment for the following reasons: voluntary (i.e., did not wish to participate, lost to follow-up before baseline assessment, unknown reason) (n = 142), not active career firefighter in target department (n = 5), relevant workers' compensation injury (n = 3), and on leave during baseline assessment period (n = 1). A total of 429 participants presented for baseline assessments. Of those, 10 were excluded for fire chief (n = 3) and missing data for current LBP from questionnaire or interview (n = 7). Thus, the final sample for analysis was n = 419, which is approximately 41.9%-46.6% (419/900-419/1,000) of the target population. For some dependent variables, the sample size was lower than 419 because of time restrictions (e.g., on-duty participants had to leave due to emergency response, technology issues (e.g., lack of cellular connection), missing information from questionnaires, contraindications to certain physical fitness/performance tests at the discretion of the clinician, and unable to complete certain assessments for other reasons. For the final sample, the point prevalence of current LBP was 19.81% (83/419).

Compared to participants without current LBP, those with current LBP had significantly worse scores for 13 of the 15 dependent variables, as illustrated in Table 1. When analyzed by age and obesity categories, scores for about one-half of the dependent variables remained significantly worse for participants with current LBP, as described below. For the other dependent variables, scores for those with current LBP appeared to be worse, though statistical significance was not reached. Overall, the Oswestry Disability Index, cardiovascular exercise frequency, and Catastrophic Injustice Experience Questionnaire scores were significantly worse for those with current LBP across all five group comparisons - all participants, age < 45 yr, age ≥ 45 y, BMI < 30 kg/m^2^ (not obese), and BMI ≥ 30 kg/m^2^ (obese). In contrast, no significant difference was observed between participants with and without current LBP for plank core muscular endurance and functional movement quality for any of the five group comparisons.

**Table 1:** Anthropometric, exercise habits, physical fitness/performance, disability/work ability, and other biopsychosocial measures in participants with and without current low back pain LBP: Low back pain. BMI: Body mass index (kg/m^2^). Body Fat: Assessed by bioelectrical impedance analysis (%). Waist Circumference: Assessed by a tape measure (cm). Exercise frequency: Assessed by a questionnaire (d/wk). Back muscular endurance: Assessed by the Ito Test (sec). Core muscular endurance: Assessed by the prone plank test (sec). Functional movement quality: Assessed by 4 tests of the functional movement screen (0-12). Back-related disability: Assessed by the Oswestry Disability Index (0-100%). Lift and carry ability: Assessed by the lift and carry sub-scale of the multidimensional task ability profile (0-200). Firefighter functional task ability: Assessed by the Firefighter Functional Task Questionnaire (0-48). Sleep quality: Assessed by the Pittsburgh Sleep Quality Index Questionnaire (0-21). Fear and fatigue: Assessed by the Fear and Fatigue Questionnaire (0-20). Catastrophic injustice experience: Assessed by the Catastrophic Injustice Experience Questionnaire (0-24). P value represents a comparison of participants with (yes) and without (no) current low back pain using one-way analysis of variance.

	Current LBP: No			Current LBP: Yes			
Variable	N	Mean	SD	N	Mean	SD	P
Anthropometric:							
BMI (kg/m^2^)	336	28.3	3.6	83	29.3	4.0	0.031
Body Fat (%)	330	21.3	5.9	83	23.6	6.0	0.002
Waist Circumference (cm)	334	93.1	9.6	83	96.4	11.2	0.007
Exercise Habits:							
Strengthening Exercise Frequency (d/wk)	336	3.1	1.6	83	2.4	1.6	0.001
Cardiovascular Exercise Frequency (d/wk)	336	3.4	1.4	83	2.7	1.5	0.000
Flexibility Exercise Frequency (d/wk)	336	2.4	1.7	83	1.9	1.6	0.006
Physical Fitness / Performance:							
Back Muscular Endurance (sec)	333	206.4	100.7	81	179.6	104.8	0.034
Core Muscular Endurance (sec)	334	149.3	64.8	81	133.6	66.5	0.052
Functional Movement Quality (0-12)	315	9.3	1.8	77	9.0	1.6	0.174
Disability/Work Ability:							
Back-Related Disability (%)	336	2.8	4.1	83	10.2	7.5	0.000
Lift and Carry Ability (0-56)	207	54.9	2.7	39	53.9	3.2	0.038
Firefighter Functional Task Ability (0-48)	335	41.7	5.8	83	39.9	5.8	0.012
Other Biopsychosocial:							
Sleep Quality (0-21)	336	6.8	3.0	83	8.0	3.5	0.004
Fear and Fatigue (0-20)	335	2.2	2.1	83	3.6	3.1	0.000
Catastrophic Injustice Experience (0-24)	335	1.0	2.2	83	3.0	4.5	0.000

Participants with current LBP were significantly older than those without current LBP (with 40.5 ± 9.2 y; without 36.9 ± 8.6 y; p = 0.0009). Specifically, 76.8% (332/419) of participants were < 45 years of age, of which 18.0% (54/332) reported current LBP. For those < 45 yr of age, compared to participants without current LBP, those with current LBP had significantly worse scores for % body fat (p = 0.0243), cardiovascular exercise frequency (p = 0.0009), Ito back muscular endurance (p = 0.0215), Oswestry Disability Index (p < 0.0001), sleep quality (p = 0.028), Fear and Fatigue Questionnaire (p = 0.0001), and Catastrophic Injustice Experience Questionnaire (p < 0.0001). Additionally, 23.2% (97/419) of participants were ≥ 45 years of age, of which 29.9% (29/97) reported current LBP. For those ≥ 45 years of age, compared to participants without current LBP, those with current LBP had significantly worse scores for strengthening exercise frequency (p = 0.0405), cardiovascular exercise frequency (p = 0.0347, flexibility exercise frequency (p = 0.0036), Oswestry Disability Index (p < 0.0001), Pittsburgh Sleep Quality Index (p = 0.028), Fear and Fatigue Questionnaire (p = 0.0001), and Catastrophic Injustice Experience Questionnaire (p = 0.0014).

Participants with current LBP had significantly higher BMI than those without current LBP, as noted in Table 1. Moreover, 71.6% (300/419) of participants had a BMI of < 30 kg/m^2^ (not obese) of which 17.3% (52/300) reported current LBP. For those with BMI < 30 kg/m^2^, compared to participants without current LBP, those with current LBP had significantly worse scores for % body fat (p = 0.0335), strengthening exercise frequency (p = 0.0273), cardiovascular exercise frequency (p = 0.0029), Oswestry Disability Index (p < 0.0001), Firefighter Functional Task Questionnaire (p = 0.03), Pittsburgh Sleep Quality Index (p = 0.0238), Fear and Fatigue Questionnaire (p = 0.0003), and Catastrophic Injustice Experience Questionnaire (p < 0.0001). Meanwhile, 28.4% (119/419) of participants had a BMI ≥ 30 kg/m^2^, of which 26.1% (31/119) reported current LBP. For those with BMI ≥ 30 kg/m^2^, compared to participants without current LBP, those with current LBP had significantly worse scores for strengthening exercise frequency (p = 0.0252), cardiovascular exercise frequency (p = 0.0087), flexibility exercise frequency (p = 0.0027), Oswestry Disability Index (p < 0.0001), Fear and Fatigue Questionnaire (p = 0.0184), and Catastrophic Injustice Experience Questionnaire (p = 0.0052).

Compared to participants without current LBP, those with current LBP had significantly greater rates of some level of back-related disability (with 96.4%, without 51.6%, p < 0.001), and perceived PDC below the firefighter job demand level of very heavy (with 51.3%, without 34.3%, p = 0.044) (Table 2). For all participants combined, 61.1% (256/419) had some level of back-related disability, 37.0% (91/246) had a perceived PDC below very heavy, and 68.3% (168/246) had some level of back-related disability and/or a perceived PDC level below very heavy.

**Table 2:** Back-related disability categories and perceived physical demand characteristics of work levels in participants with and without current low back pain LBP: Low back pain Back-related disability - ODI: Assessed by the Oswestry Disability Index, which is scored from 0% to 100% with disability categories as noted in the table. Work ability - perceived PDC level: Perceived physical demand characteristics of work level assessed by the Multidimensional Task Ability Profile, which is scored from 0 to 200 with PDC categories as noted in the table. P value represents a comparison of participants with (yes) and without (no) current low back pain using chi-square analysis.

	Current LBP: No		Current LBP: Yes		
Variable	N	%	N	%	P
Back-Related Disability - ODI:					0.000
None (0%)	162	48.2%	3	3.6%	
Minimal (1-20%)	171	50.9%	73	88.0%	
Moderate (21-40%)	3	0.9%	7	8.4%	
Total	336		83		
Work Ability - Perceived PDC Level:					0.044
Very Heavy (200)	136	65.7%	19	48.7%	
Heavy (192-199)	66	31.4%	16	41.0%	
Medium (164-191)	5	2.4%	4	10.3%	
Light (100-163)	1	0.5%	0	0.0%	
Total	207		39		

Table 3 depicts the Pearson product-moment correlations among the dependent variables. Overall, 81.7% (98/120) of the pairwise comparisons showed significant relationships between the variables. Of those, 51.0% (50/98) had weak correlations with R values ranging from 0.20 to 0.39 [51].

**Table 3:** Correlations among the variables Values are Pearson product-moment correlation coefficients. * indicates p < 0.05 for the pairwise comparison. † indicates p < 0.01 for the pairwise comparison. ‡ indicates p < 0.001 for the pairwise comparison. Age. BMI: Body mass index (kg/m^2^). BF: Body fat (%). Waist: Waist circumference (in). EF-S: Strengthening exercise frequency (d/wk). EF-C: Cardiovascular exercise frequency (d/wk). EF-F: Flexibility exercise frequency (d/wk). Ito: Back muscular endurance (sec). Plank: Core muscular endurance (sec). FMQ: Functional movement quality (0-12). ODI: Back-related disability from the Oswestry Disability Index (%). MTAP: Lift and carry sub-scale from the Multidimensional Task Ability Profile (0-56). FFTQ: Firefighter Functional Task Questionnaire (0-48). PSQI: Pittsburgh Sleep Quality Index (0-21). FAFQ: Fear and Fatigue Questionnaire (0-20). CIEQ: Catastrophic Injustice Experience Questionnaire (0-24).

Variable	Age	BMI	BF	Waist	EF-S	EF-C	EF-F	Ito	Plank	FMQ	ODI	MTAP	FFTQ	PSQI	FAFQ	CIEQ
Age	1															
BMI	0.15*	1														
BF	0.35‡	0.78‡	1													
Waist	0.32‡	0.89‡	0.80‡	1												
EF-S	-0.35‡	-0.24‡	-0.39‡	-0.37‡	1											
EF-C	-0.16‡	-0.24‡	-0.30‡	-0.26‡	0.53‡	1										
EF-F	-0.09	-0.21‡	-0.23‡	-0.21‡	0.34‡	0.33‡	1									
Ito	-0.01	-0.26‡	-0.29‡	-0.25‡	0.13†	0.08	0.18‡	1								
Plank	-0.10*	-0.40‡	-0.47‡	-0.47‡	0.35‡	0.20‡	0.16†	0.49‡	1							
FMQ	-0.13*	-0.31‡	-0.36‡	-0.30‡	0.22‡	0.13*	0.26‡	0.24‡	0.26‡	1						
ODI	0.32‡	0.06	0.17‡	0.11*	-0.24‡	-0.22‡	-0.04	-0.08	-0.11*	-0.08	1					
MTAP	-0.18†	0.03	-0.11	-0.02	0.12	0.01	-0.04	0.03	0.10	0.06	-0.42‡	1				
FFTQ	-0.23‡	-0.12*	-0.24‡	-0.19‡	0.32‡	0.26‡	0.17‡	0.14†	0.19‡	0.13†	-0.17‡	0.34‡	1			
PSQI	0.10*	0.08	0.11*	0.06	-0.09	-0.13†	-0.09	-0.11*	-0.11*	-0.17‡	0.38‡	-0.23‡	-0.06	1		
FAFQ	0.21‡	0.21‡	0.31‡	0.25‡	-0.32‡	-0.32‡	-0.23‡	-0.17‡	-0.25‡	-0.17‡	0.46‡	-0.21‡	-0.17‡	0.46‡	1	
CIEQ	0.16‡	0.12*	0.16‡	0.15†	-0.20‡	-0.22‡	-0.17‡	-0.15†	-0.18‡	-0.20‡	0.49‡	-0.20†	-0.06	0.39‡	0.60‡	1

## Discussion

The present study indicated that nearly one-fifth of full-duty career firefighters without documented work restrictions reported having current LBP, and these individuals had deficits in several modifiable biopsychosocial factors across five health domains - anthropometric, exercise habits, physical fitness/performance, disability/work ability, and other biopsychosocial measures. In addition, this study demonstrated that certain health assessments can distinguish between participants with and without current LBP. These findings can help guide future research and implementation efforts in the fire service designed to improve performance, resiliency, work readiness, recovery, and quality of life, as well as reduce impairment, disability, and absenteeism and increase presenteeism.

The present study found a slightly higher point prevalence of current LBP in firefighters compared with a published report of adults in the general population that observed point prevalences ranging from 1.0% to 58.1% (mean: 18.1%, median: 15.0%) [29]. For firefighters, the point prevalence of current LBP in the present study was similar to the 20.1% (53/264) rate found in previous research (unpublished data from U.S. Department of Homeland Security grant # EMW-2013-FP-00723). The present study and previous research had similar eligibility criteria (full duty career without documented work restrictions) and used the same question to assess current LBP, yet were conducted in different regions of the U.S. with different purposes (quality improvement implementation project vs randomized controlled trial).

In contrast, the present study found a lower point prevalence of current LBP in firefighters compared with published reports from three U.S. fire departments, which ranged from 30.2% [16], 43.9% [17], to 55% [1]. Possible explanations for the observed differences between the present study and other reports include the following:

(1) Differences in and uncertainty of eligibility criteria of the participants surveyed in the other reports;

(2) It is uncertain if the other reports enrolled only full-duty career firefighters without documented work restrictions;

(3) It is uncertain how the low back region was defined in the other reports;

(4) A different question was used to identify current LBP compared to the present study, or the specific question used for this purpose was not provided;

(5) Participants in the present study who were excluded from analysis (n = 10) may have had higher rates of current LBP than those included. Seven were excluded because of missing data for current LBP from questionnaires or interviews; thus, it is not possible to assess current LBP rates for these participants. Three were excluded for being fire chiefs. One of these three had current LBP, which would not substantially impact the rate of current LBP observed in the assessed sample.

(6) Those from the target population who did not participate may have had higher rates of current LBP than those who participated. The participants of this study were drawn from a sample of convenience. Thus, participation bias or selection bias may have occurred, which could have impacted the observed rates of current LBP. However, data about this measure in the entire target population were unavailable for comparison.

Based on the present study and previous studies, it is evident that a considerable number of full-duty firefighters are working with current LBP. This finding is consistent with research suggesting that firefighters under-report LBP to departmental administration and continue to work despite the presence of LBP [4]. Plausible explanations for why firefighters continue to work with LBP include the following: (1) financial benefits: overtime work can be a substantial contributor to compensation and those out of work or on restricted duty are unable to perform overtime work; (2) job security [4]: some may fear that reporting LBP could result in retribution or placement on restricted duty; (3) dedication to occupation [4]: firefighters wish to serve the community in the capacity they signed up for. They do not want to be placed on modified duty, such as sitting at a desk performing office work; and (4) perceptions about LBP severity [4]: some may perceive that their LBP is not severe enough to justify reporting to departmental administration or seeking health care.

The present study indicated that firefighters working with current LBP have deficiencies in several modifiable biopsychosocial factors across various health domains. Overall, those with current LBP had significantly worse scores for 13 of the 15 assessments compared with their counterparts without current LBP. While the deficits observed for the entire cohort generally held up when analyzed by age and obesity categories, statistical significance was lost for some of the variables. One explanation for the loss of statistical significance is that the sample size was not large enough to detect group differences when stratified by age and obesity categories. Another explanation is that the assessed health variables impact firefighters differently among these categories.

The present study found that performance on the back muscular endurance test was significantly worse in participants with current LBP, which is consistent with previous research on back endurance in firefighters with a lifetime history of LBP [3]. On the contrary, the two other assessed physical fitness tests (core muscular endurance, functional movement quality) were unrelated to current LBP status, which may question the use of the prone plank test and FMS for LBP risk screening in this population.

Another important finding of the present study is that a large percentage of participants reported working with some level of back-related disability and/or perceived PDC level below the required very heavy job demands of firefighters, and these deficits were worse in those with current LBP. Regular service full-duty firefighters are typically expected to perform occupational activities at the very heavy PDC level, which requires occasional lifting and carrying greater than 100 pounds (45 kg) [19]. Moreover, regardless of age, anthropometric characteristics, and physical fitness status, all full-duty personnel are expected to perform at this level. For clinical decision-making, perceptions related to disability and workability/PDC should be compared with actual physical fitness and performance tests, and discrepancies between the two should be considered within the plan of care. In the present study, perceived back-related disability and perceived lift and carry ability were moderately correlated with each other (R = -0.42, p < 0.001). However, neither of these variables was significantly correlated with back muscular endurance and functional movement quality, and back-related disability was only very weakly correlated with core muscular endurance (R = -0.11, p < 0.05) (Table 3). Future research is needed to examine how the interactions between perceived ability and actual physical performance affect work-related outcomes and quality of life.

The strengths of the present study are a large sample size, all participants were full-duty career firefighters without documented work restrictions, and a wide range of modifiable biopsychosocial factors across important health domains were assessed. This study also has limitations. First, it was a cross-sectional design; thus, establishing the cause-effects of the uncovered relationships was not feasible. Moreover, due to its specific aim and exploratory nature, other important work-related measures, such as lost work time (absenteeism), presenteeism, and costs, as well as potential LBP covariates and comorbidities, were not examined. Additionally, LBP severity, specific regions of spinal pain (e.g., sacroiliac joint, disc, facet), and clinical diagnoses were not assessed in the present study - all of which potentially could affect the study's observations. Furthermore, possible gender differences in the relationships between current LBP and the assessed variables were not explored. Preliminary findings have suggested that targeted interventions for firefighters may help improve deficits in some of the present study's assessments [25], but it is unknown if such improvements could be achieved in those with current LBP through evidence-based interventions. Lastly, the minimal clinically important differences (MCIDs) of the study assessments have not been fully characterized in firefighters. Future research is needed to address these limitations.

The present study's findings may be useful for various individuals and groups engaged with the fire service, such as employees at risk for LBP, the communities they serve, administrators, and the interprofessional team of healthcare specialists who manage work-related LBP and other musculoskeletal injuries for this population. The assessments utilized in this study are clinically meaningful across important health domains for musculoskeletal disorders, pragmatic, and non-invasive and can be used in a wide variety of fire service settings without the need for complex equipment or large space requirements. Moreover, the interrelationships observed among these health domains provide additional justification for implementing a comprehensive biopsychosocial approach for the prevention and treatment of musculoskeletal injuries in the fire service. These assessments can serve as components of the industrial athlete model to manage high-risk workers [52] and wellness-fitness programs designed to meet the needs of firefighters with current LBP. For example, the multi-faceted assessments may be used to help guide triage decisions for those who need further diagnostic work-up. If administered serially to document progress and outcomes, they can be critical for prescribing client-centered preventive programs and therapies. Another potential application of the results of this study is to compare perceptions of work ability (perceived PDC) with actual physical performance abilities in firefighters with current LBP and address discrepancies accordingly. It is plausible that large discrepancies between perceived and actual abilities may indicate clinically relevant factors for delayed recovery (e.g., harboring a chronic injury), stoic behaviors, and not reporting a claim or disability. Additionally, these assessments could be useful to help identify firefighters with current LBP who may need early and appropriate rehabilitation, which consists of education about expectations for recovery, exercise, and manual therapy [20].

## Conclusions

A cross-sectional study was conducted using baseline assessments from 419 full-duty career firefighters without documented work restrictions who were enrolled in a regional wellness initiative in Southern California, USA. Nearly one-fifth of participants reported having current LBP, and these individuals had deficits in several modifiable biopsychosocial factors across five health domains - anthropometric, exercise habits, physical fitness/performance, disability/work ability, and other biopsychosocial categories. This study also found that a large percentage of participants are working with some level of back-related disability and/or perceived physical demand characteristics of work level below the required very heavy job demands for this occupation. These findings can help guide future research and implementation efforts in the fire service designed to improve performance, resiliency, work readiness, recovery, and quality of life, as well as reduce impairment, disability, and absenteeism and increase presenteeism.
